# Association of HIV-1 Infection and Antiretroviral Therapy With Type 2 Diabetes in the Hispanic Population of the Rio Grande Valley, Texas, USA

**DOI:** 10.3389/fmed.2021.676979

**Published:** 2021-07-05

**Authors:** Juan Carlos Lopez-Alvarenga, Dora A. Martinez, Alvaro Diaz-Badillo, Liza D. Morales, Rector Arya, Christopher P. Jenkinson, Joanne E. Curran, Donna M. Lehman, John Blangero, Ravindranath Duggirala, Srinivas Mummidi, Ruben D. Martinez

**Affiliations:** ^1^Department of Human Genetics, South Texas Diabetes and Obesity Institute, School of Medicine, University of Texas Rio Grande Valley, Edinburg, TX, United States; ^2^Valley AIDS Council, Harlingen, TX, United States; ^3^Department of Medicine, University of Texas Health San Antonio, San Antonio, TX, United States

**Keywords:** South Texas, AIDS, type 2 diabetes, HIV, Mexican Americans, antiretroviral treatment, Rio Grande Valley, obesity

## Abstract

The Rio Grande Valley (RGV) in South Texas has one of the highest prevalence of obesity and type 2 diabetes (T2D) in the United States (US). We report for the first time the T2D prevalence in persons with HIV (PWH) in the RGV and the interrelationship between T2D, cardiometabolic risk factors, HIV-related indices, and antiretroviral therapies (ART). The PWH in this study received medical care at Valley AIDS Council (VAC) clinic sites located in Harlingen and McAllen, Texas. Henceforth, this cohort will be referred to as Valley AIDS Council Cohort (VACC). Cross-sectional analyses were conducted using retrospective data obtained from 1,827 registries. It included demographic and anthropometric variables, cardiometabolic traits, and HIV-related virological and immunological indices. For descriptive statistics, we used mean values of the quantitative variables from unbalanced visits across 20 months. Robust regression methods were used to determine the associations. For comparisons, we used cardiometabolic trait data obtained from HIV-uninfected San Antonio Mexican American Family Studies (SAMAFS; *N* = 2,498), and the Mexican American population in the National Health and Nutrition Examination Survey (HHANES; *N* = 5,989). The prevalence of T2D in VACC was 51% compared to 27% in SAMAFS and 19% in HHANES, respectively. The PWH with T2D in VACC were younger (4.7 years) and had lower BMI (BMI 2.43 units less) when compared to SAMAFS individuals. In contrast, VACC individuals had increased blood pressure and dyslipidemia. The increased T2D prevalence in VACC was independent of BMI. Within the VACC, ART was associated with viral load and CD4+ T cell counts but not with metabolic dysfunction. Notably, we found that individuals with any INSTI combination had higher T2D risk: OR 2.08 (95%CI 1.67, 2.6; *p* < 0.001). In summary, our results suggest that VACC individuals may develop T2D at younger ages independent of obesity. The high burden of T2D in these individuals necessitates rigorously designed longitudinal studies to draw potential causal inferences and develop better treatment regimens.

## Introduction

Current antiretroviral therapies (ART) have dramatically improved the quality of life and increased the life span of persons with HIV (PWH). However, when compared to the general population, PWH have an increased incidence of common age-associated degenerative diseases, including metabolic disorders such as type 2 diabetes (T2D) and cardiovascular disease (CVD). The Hispanic/Latino community comprises ~23% of the PWH and 27% of the ~36,400 newly diagnosed HIV cases in the US in 2018. A recent study estimated that the median Latino disparity in the HIV prevalence to be 2.4 (1). Hispanics also have a higher prevalence of cardiometabolic risk factors that predispose them to T2D when compared to non-Hispanic whites (NHW). For example, Lorenzo et al. compared the non-HIV infected Mexican American population with non-Hispanic whites, using Matsuda and insulinogenic indexes to assess insulin resistance. They showed that Mexican Americans have an odds ratio of 2.3 (95%CI 1.5, 3.34) for insulin resistance (2). Thus, HIV-1 infected Hispanics are at an even greater risk of developing T2D and T2D-related complications. However, few studies have systematically examined the incidence of these metabolic disorders in HIV-infected Hispanics, especially in the U.S.-Mexico border region who encounter unique socio-cultural, economic, migration-related challenges.

Prior to the introduction of the new generation of ART, the incidence of diabetes was found to be 4-fold higher in HIV-positive men when compared to HIV-negative men in the Multicenter AIDS Cohort Study (MACS) (3). While the recent ART regimens have less dramatic effects, there appears to be growing evidence of increasing T2D prevalence in HIV-positive individuals in the US. In a recent large cross-sectional study (*N* = 17,946,580) that included 90,900 persons with HIV (60.8% male), Birabaharan et al. found that the overall prevalence of T2D in the HIV group was 22.1% (*N* = 20,080) which is much higher than the T2D prevalence in the US population which is 14.9%. Notably, the prevalence of T2D was higher in the group classified as “others,” which includes the Hispanic population (27.6 vs. 21.3% in the white population). Nansseu et al. performed a systematic review and metanalysis of 44 studies conducted across the globe and projected that in a “cohort of 1,000 PWH starting ART, about 50 may have T2D after 3.7 years of follow-up while around 457 may develop prediabetes within the same period” (4). Other metabolic abnormalities that predispose HIV-infected individuals to T2D have also been reported with currently used ART regimens. A recent large-scale study (*N* = 14,084) showed that 22% of lean PWH individuals became overweight just after 3 years on ART (5). A similar trend was observed in overweight people who became obese. In another study, Sax et al. performed a pooled analysis of weight gain in eight randomized controlled clinical trials of treatment-naïve PWH (*N* = ~5,000) and found that demographic factors (e.g., female, black), HIV related factors at the start of the treatment (e.g., low CD4, high viral load), and ART regimen used may play a role in weight gain (6). Thus, the contributions to weight gain in ART-treated PWH could be multifactorial, and both HIV/ART and traditional risk factors could lead to increased weight gain and metabolic risk in PWH. This is of great concern as a majority of MAs are overweight or obese with increased genetic susceptibility to T2D and other metabolic diseases and, when infected with HIV, may have a poorer response to T2D medications compared to non-Hispanic whites (7). The increased risk to T2D could be attributed to a family history of diabetes, impaired glucose tolerance, obesity, insulin resistance, and genetic factors.

The Rio Grande Valley (RGV) of South Texas has one of the highest prevalence rates of obesity and T2D in the US (8, 9). Thus, the intersection of HIV-1 infection and metabolic disorders such as T2D impose enormous health burden in the PWH in the RGV. However, there is a lack of focused studies in the PWH in the RGV especially with regard to complications arising due to ART and their management. This is important as the Hispanics in the RGV, a majority of whom are Mexican Americans (10), face unique socio-economic challenges and cultural practices and have a genetic predisposition to develop metabolic diseases. Here, we report for the first time the prevalence of T2D and its associated risk factors in PWH in the RGV, who are all enrolled at a single clinic, and contrast them with the HIV-negative Mexican Americans in the US and South Texas. We also report the interrelationship between cardiometabolic risk factors, T2D risk, HIV viral load, various immunological parameters, and treatment regimens in this cohort.

## Methods

### The Valley AIDS Council Cohort

All the individuals received medical care under the supervision of two physicians at the two Valley AIDS Council (VAC) clinic sites located in Harlingen and McAllen, TX. The RGV mainly comprises of four counties (Cameron, Hidalgo, Starr, and Willacy) that are in the Southeastern region of Texas bordering Mexico and the overall percentage of Hispanics is ~90% (10). Henceforth, this cohort of PWH in the RGV will be referred to as the Valley AIDS Council Cohort (VACC). The VAC is the only Ryan White funded agency providing medical care and supportive services for people living with HIV/AIDS in regions of Texas that are south and east of San Antonio, TX, including the cities of Corpus Christi, McAllen, Harlingen and Brownsville. In 1994, a Ryan White Title III grant enabled the agency to provide primary health care for HIV/AIDS patients in this region, as a primary healthcare outpatient clinic. The VAC has provided continuous services, including HIV testing and prevention efforts, since its start in 1987 and currently serves a large patient population (~2,000).

### Data Collection

#### Valley AIDS Council Cohort

All analyses were conducted using deidentified data extracted from a retrospective database using protocols approved by the Institutional Review Board (IRB) of the University of Texas Rio Grande Valley (UTRGV). The VAC houses four servers at the Westbrook Clinic in Harlingen that work in tandem to run eClinicalWorks (Electronic Medical Records). The VAC also uses ARIES (AIDS Regional Information and Evaluation System) and CMBHS (Clinical Management for Behavioral Health Services), both of which are State databases where patient information is entered as part of the clinical routine. A total of 1,827 records with unique IDs were abstracted from medical charts which were documented between November 2017–October 2019. The following variables were extracted from the database: demographic (age, sex, and marital status), anthropometric [height, weight, and body mass index (BMI)] and cardiometabolic traits (blood pressure, fasting glucose, HbA1c, and lipids), and HIV-related virological and immunological indices (viral load, absolute and relative CD4+ and CD8+ T cell counts, and CD4/CD8 ratio). For analysis, the WHO obesity classification system based on BMI (computed with kg/m^2^ and categorized as normal weight <25, overweight between 25 and 30, obesity class I between 30 and 35, class II between 35 and 40 and class III >40) was used (11). We used the ADA definition for defining T2D, which includes one of the following conditions: a clinical history of T2D, a fasting glucose ≥ 126 mg/dL, or HbA1C > 6.4% (12).

#### San Antonio Mexican American Family Studies and NHANES 2018 Data

For comparison with the VACC, we used data from known HIV-1 negative cohorts with Mexican American ancestry, namely the San Antonio Mexican American Family Studies (SAMAFS) and a subset of National Health and Nutrition Examination Survey (NHANES) individuals with reported Mexican American ancestry, henceforth referred to as the Hispanic Health and Nutrition Examination Survey (HHANES). The SAMAFS refers to the combination of two San Antonio-based family studies called the San Antonio Family Heart Study (SAFHS) (13, 14) and the San Antonio Family Diabetes and Gallbladder Study (SAFDGS) (15, 16). The SAFHS included 1,431 individuals in 42 large pedigrees at baseline from low-income Mexican Americans selected at random without regard to the presence or absence of disease. The SAFDGS enrolled 579 individuals distributed across 32 large pedigrees at baseline. These families were ascertained by probands with T2D who were low-income Mexican American men and women. Both study-specific protocols were approved by the IRBs of the University of Texas Health San Antonio (UTHSA) and the UTRGV. The major objective of SAMAFS was to investigate the genetics of complex diseases such as T2D, CVD, obesity and their related traits such as BMI, glucose, insulin, lipids, and blood pressure measures using standard protocols (14, 17–20). The T2D status was diagnosed according to the criteria of the World Health Organization: fasting glucose levels ≥ 126 mg/dL and/or 2-h glucose levels ≥ 200 mg/dL (21) and/or the American Diabetes Association criteria: fasting glucose levels ≥ 126 mg/dL (22). Participants who did not meet these criteria but who reported that they were under treatment with either oral antidiabetic agents or insulin and who gave a history of diabetes were also considered to have T2D. The SAMAFS participants have been followed in a mixed longitudinal fashion, up to a maximum of five visits. The original families have been expanded through additional recruitment and eight new families were added (i.e., SAFDGS) over the years. The data used for this study correspond to those measured at the last clinic examination, and individuals with T2D status information and associated cardiometabolic data were considered for the analysis (*N* = 2,498). The NHANES data were derived from the surveys conducted during 2017–2018. The HHANES data included measured glucose, HbA1c, blood pressure, triglycerides, and HDL-C for comparison with VACC. The T2D status for the individuals enrolled in NHANES was obtained from the publicly available databases (23).

### Statistical Analyses

We used 1,827 entries from VACC for cross-sectional data analyses. We used mean values of the quantitative variables from unbalanced visits across 20 months. Continuous variables were aggregated to obtain mean values and if necessary, were transformed to obtain normal distributions. An illustrative example of square root transformation to normalize CD4 counts is shown in Supplementary Figure 1. Descriptive statistics were used to evaluate the demographic and biochemical variables related to T2D and cardiometabolic risk factors in the VACC. Student *t*-tests, adjusted for variance, were performed to contrast PWH with and without T2D. Robust regression methods with robust Huber-White sandwich method HC3 (an approximation to a jackknife estimator using squared residuals divided by the square of 1-h, the letter “h” represents the leverage values in the “hat” matrix) were used to determine the associations between various immunological and HIV-related indices (24). The association between the treatment regimen and T2D, and various cardiometabolic risk factors, i.e.,: LDL-C higher than 110 mg/dL, systolic blood pressure > 140 mmHg, diastolic blood pressure > 90 mmHg, and HDL-C adjusted by sex (females <50 mg/dL and males <40 mg/dL) were performed using logistic regression to obtain odds ratios (ORs) (95% CI). Each model was adjusted by sex, age, WHO BMI categories, viral loads (<50 copies/mL, between 50 and 1,000 copies/mL; and >1,000 copies/mL), and absolute CD4+ counts (<500 cells/mm^3^, between 500 and 900 cells/mm^3^, and >900 cells/mm^3^). A backward stepwise regression with *p*-values <0.20 was performed. The variables collected from the VACC were compared with the cardiometabolic trait data from SAMAFS (*N* = 2,498) after adjusting for pedigree structure and the HHANES data (*N* = 5,989) using robust regressions. Descriptive statistics such as means, standard deviations, and frequencies were used to compare VACC, SAMAFS, and HHANES data. ANOVA with robust HC3 method was used for comparing the continuous variables between the three cohorts after adjusting for pedigree structure. All analyses were performed with STATA/SE 16.0 (Stata Corp. LLC. College Station TX. USA).

## Results

### Descriptive Statistics of the VACC

We extracted a total of 2,959 entries from the patient registries in the VAC database, out of which 1,827 entries were suitable for further analysis. All entries were from patient registries in the VAC database entered between November 2017 to October 2019. However, not all variables were available for each registry, and the sample size varied from trait to trait based on the available information. Hence, maximal information was provided for a given trait by using the available data for description. Please note that the use of a value was conditioned by the presence of other variables (for example, the descriptive tables for age require the presence of sex and age from a specific sample; meanwhile, the simple description of sex in the same sample was not conditioned by the presence of age) giving slightly different mean values from descriptive tables and the text. The median number of visits was 8 (Q1 5.3, Q3: 9.2) for each individual. The following data were available: sex (1,362/1,707; 80% male) and T2D status (917/1807; 51%). The mean (SD) age was 42.1 (12.7) years old, BMI 29.4 (6.1), HDL-C 44.5 (12.7) mg/dL, LDL-C 96 (25) mg/dL, triglycerides 176 (121.6) mg/dL. The most common treatment was integrase stand transfer inhibitors (INSTIs) in any combination was 64% (918/1,425) of the PWH in the VACC. The most frequent combination was INSTI+Nucleoside/Nucleotide reverse transcriptase inhibitors (NRTI) in 22% (317/1,425) of the individuals. Other treatment regimens used were combinations containing Protease inhibitors (PIs) and NRTI + Non-nucleoside reverse transcriptase inhibitors (NNRTI). The absolute CD4+ counts were 589 (315) cells/mm^3^, absolute CD8+ counts 909 (427) cells/mm^3^, and the mean CD4/CD8 ratio was 0.76 (0.49). The frequency of BMI categories as defined by the WHO (11) were as follows: normal 375 (23%), overweight 626 (38%), obesity class I were 393 (24%), class II were 147 (9%), and class III were 97 (6%). Males had higher blood pressure and lower BMI, HDL-C, CD4/CD8 ratio compared with females. Table 1 summarizes the descriptive conditional statistics of the demographic and clinical data and comparative analysis between the sexes in the VACC.

**Table 1:** General description of the Valley AIDS Council Cohort (VACC).

**Variables**	**All Individuals**		**Females**		**Males**		***p*-value**
	***N***	**Mean ± SD**	***N***	**Mean ± SD**	***N***	**Mean ± SD**	
Age (years)	1,701	42.5 (12.6)	342	44.6 (12.3)	1,359	42 (12.7)	<0.001
BMI	1,637	29.4 (6.1)	329	31.8 (7.1)	1,308	28.8 (5.6)	<0.001
SBP^¶^ (mmHg)	1,696	128.8 (13.2)	341	124.6 (15.5)	1,355	129.8 (12.3)	<0.001
DBP (mmHg)	1,696	80.6 (7.9)	341	78 (7.8)	1,355	81.3 (7.7)	<0.001
Triglycerides (mg/dL)	1,438	177.6 (123.3)	301	168.4 (155.9)	1,137	180 (113)	0.144
HDL-C (mg/dL)	1,437	44.7 (12.4)	301	49.7 (12.1)	1,136	43.3 (12.1)	<0.001
LDL-C (mg/dL)	1,425	97.7 (28.1)	299	98.7 (28.9)	1,126	97.4 (27.9)	0.506
Abs CD4 per mm^3^	1,392	597.9 (308.4)	296	638.7 (302.4)	1,096	587 (309.2)	0.01
Abs CD8 per mm^3^	1,349	900.9 (401.8)	288	803.9 (366.8)	1,061	927.2 (407)	<0.001
CD4%	1,391	27.7 (11.1)	296	31.5 (11.2)	1,095	26.7 (10.8)	<0.001
CD8%	1,350	42.8 (12.5)	288	40.2 (12.3)	1,062	43.5 (12.4)	<0.001
CD4/CD8	1,417	0.77 (0.49)	299	0.95 (0.55)	1,118	0.73 (0.46)	<0.001

*The table shows total/combined and sex-wise mean values of the demographic and clinical characteristics*.

¶ *SBP, systolic blood pressure; DBP, diastolic blood pressure; Abs, absolute values*.

### HIV-1 Related Indices and Their Relation to VACC Metabolic Traits

**Figure 1:**
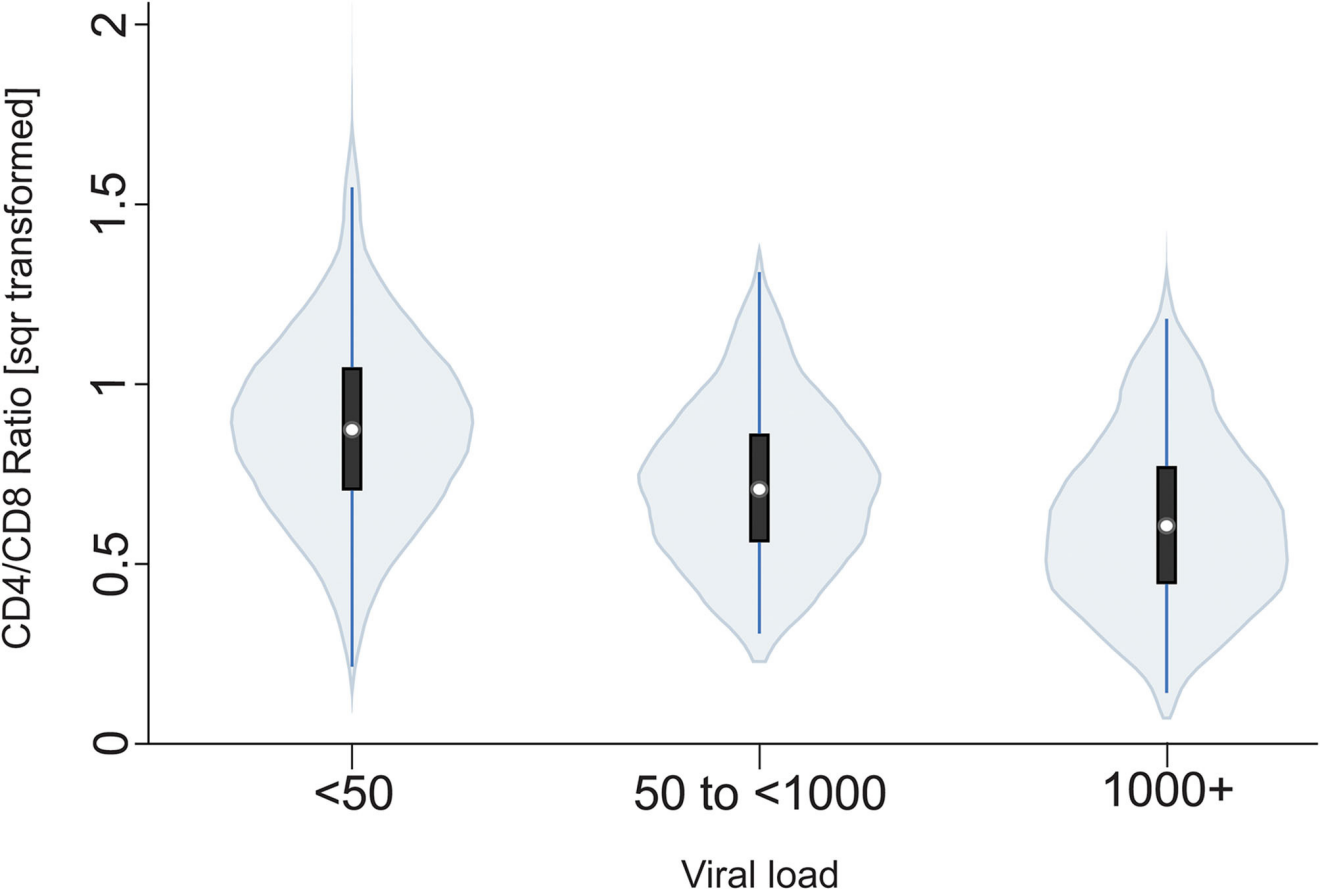
Violin plot of the relationship between CD4/CD8 ratio and viral load. Square root transformed CD4/CD8 ratios are shown on the y-axis which decrease as a function of increasing viral loads (<50 copies, 50–1,000 copies and >1,000 copies) shown on x-axis [*b* = −0.13 (se 0.01), *p* < 0.001].

**Figure 2:**
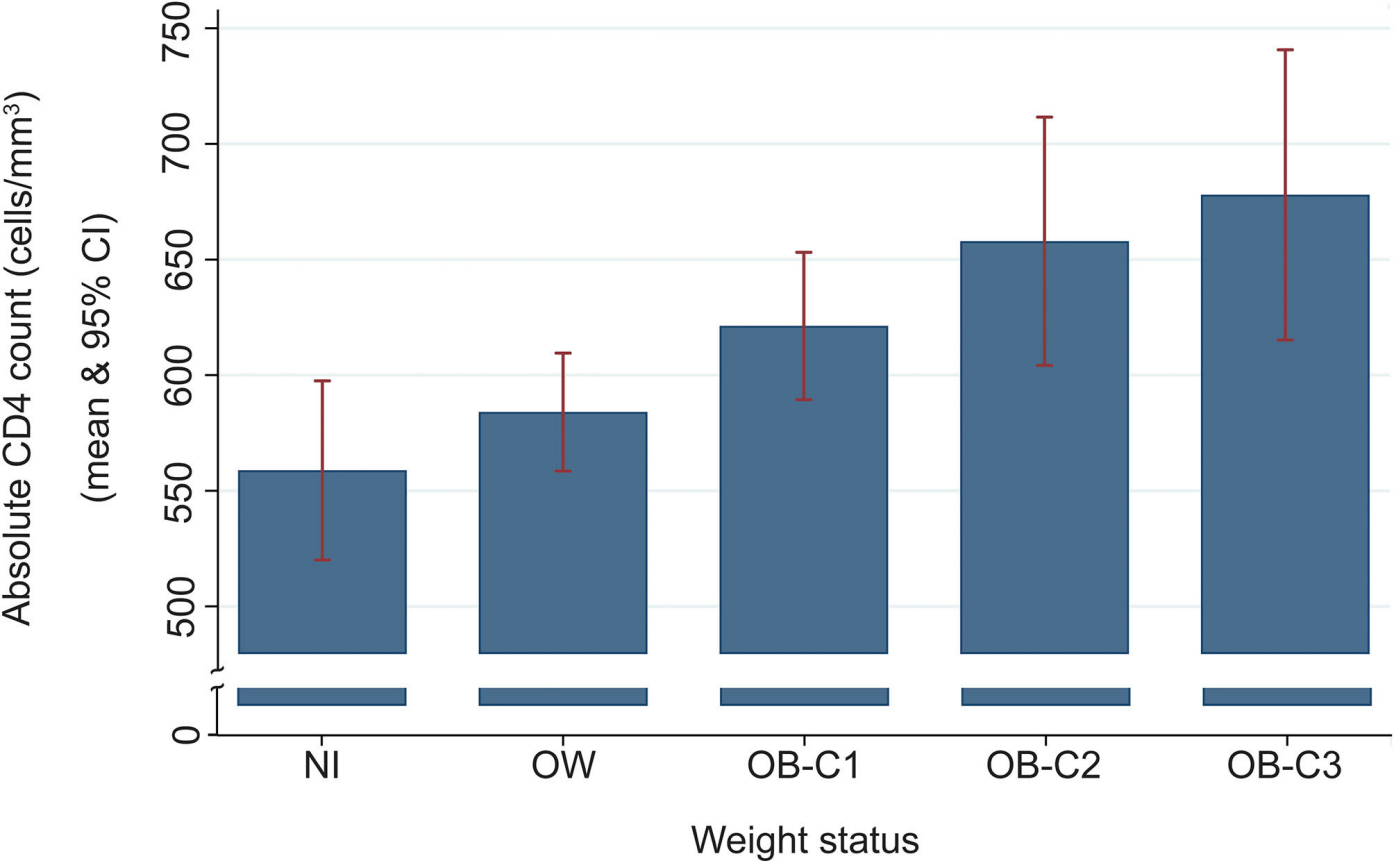
A histogram depicting an increase of absolute CD4 lymphocytes with an increase of body weight. The regression with CD4+ T cell counts (square root transformed) was *b* = 0.48 (robust HC3 se = 0.16), *p* = 0.004. NW, normal weight; OW, overweight; OB-C1, obesity-category 1; OB-C2, obesity-category 2; and OB-C3, obesity category 3.

We then evaluated several HIV-1 related indices in the VACC and their relationship to various metabolic traits. Eighty percent of the VACC individuals had viral loads lower than 50 copies/mL (*N* = 1,182), six percent showed viral loads between 50 and 1,000 copies/mL (*N* = 92), and fourteen percent showed >1,000 copies/mL (*N* = 212). The virus load was an independent predictor of CD4+ counts, CD8+ counts, % CD4, % CD8, and CD4/CD8 ratio adjusted by age, sex, presence of T2D, lipid concentration, and duration of enrollment in the clinic (Table 2). There was no difference in viral loads between men and women (*p* = 0.34). Figure 1 shows a violin plot depicting the relationship between CD4/CD8 ratio and viral load, and as expected, higher viral loads were negatively associated with CD4/CD8 ratios. We also found that increasing BMI was positively associated with increasing CD4+ cell counts (Figure 2). T2D was found in 51% of the VACC individuals (917/1,807), with no differences by sex (females OR = 0.89, 95%CI: 0.68, 1.13; *p* = 0.306). However, for example, participants with T2D had higher CD8+ cell % (Cohen-*d* 25.6%, *p* < 0.001) and less LDL-C (Cohen-*d* 0.24, *p* < 0.001) compared to those without T2D (Table 3).

**Table 2:** Factors associated with laboratory immunological parameters in the VACC^¶^.

**Predictors**	**Abs CD4+**	**Abs CD8+**	**CD4%**	**CD8%**	**CD4/CD8 ratio**
HIV load (cat)^†^	−2.960 (0.259)^*^	0.047 (0.019)^*^	−4.624 (0.418)^*^	6.088 (0.524)^*^	−0.131 (0.009)^*^
Sex	−0.927 (0.427)^*^	0.147 (0.032)^*^	−4.244 (0.759)^*^	2.855 (0.797)^*^	−0.108 (0.019)^*^
BMI	0.481 (0.165)^*^	0.028 (0.011)^*^	0.305 (0.275)	−0.256 (0.291)	0.007 (0.007)
T2D	−0.552 (0.347)	−0.001 (0.024)	−0.268 (0.599)	1.505 (0.659)^*^	−0.018 (0.014)
Time attendance	0.185 (0.079)^*^	−0.003 (0.006)	0.435 (0.128)^*^	−0.232 (0.155)	0.011 (0.003)^*^
Age	−0.066 (0.014)^*^	−0.006 (0.001)^*^	−0.060 (0.023)^*^	−0.061 (0.027)^*^	−0.001 (0.001)
LDL-C	0.013 (0.006)^*^	0.001 (0.001)	0.01 (0.01)	−0.011 (0.012)	0.001 (0.001)
Triglycerides	0.002 (0.002)	0.001 (0.001)^*^	−0.005 (0.003)	0.003 (0.003)	−0.001 (0.001)

*Data are estimated coefficients (SEM)*.

† *The HIV load was divided into three categories: <50, between 50 and 1,000, and >1,000*.

¶ *Abs, absolute values*.

* *p-values < 0.05. VACC, Valley AIDS Council Cohort*.

**Table 3:** Descriptive statistics of the VACC individuals stratified by T2D status^¶^.

**Variables**	**All Individuals**		**No T2D**		**T2D**		***p*-value**
	***N***	**Mean ± SD**	***N***	**Mean ± SD**	***N***	**Mean ± SD**	
Age (years)	1,500	43 (12.7)	759	42.1 (12.3)	741	43.8 (13)	0.01
BMI	1,428	29.5 (6.1)	728	29.9 (6.3)	700	29.1 (5.9)	0.021
SBP (mmHg)	1,466	129.2 (12.9)	744	129.1 (12)	722	129.3 (13.8)	0.804
DBP (mmHg)	1,466	80.8 (7.6)	744	81.1 (7.3)	722	80.5 (7.8)	0.095
Triglycerides (mg/dL)	1,423	177.8 (124.1)	598	173.1 (99.6)	825	181.3 (139.1)	0.218
HDL-C (mg/dL)	1,422	44.5 (12.7)	598	45.1 (12.3)	824	44.1 (12.9)	0.147
LDL-C (mg/dL)	1,411	96.7 (28.4)	594	100.6 (27.5)	817	93.9 (28.7)	<0.001
CD4/CD8	1,403	0.75 (0.49)	588	0.8 (0.49)	815	0.72 (0.49)	0.003
Abs CD4 per mm^3^	1,402	587.9 (316.7)	587	618.1 (314.2)	815	566.2 (317)	0.002
Abs CD8 per mm^3^	1,380	910.4 (429.9)	577	898.6 (408.4)	803	918.8 (444.9)	0.389
CD4%	1,401	27.3 (11.2)	586	28.2 (10.8)	815	26.6 (11.5)	0.008
CD8%	1,380	43.6 (12.8)	577	41.7 (11.6)	803	44.9 (13.4)	<0.001

¶ *VACC, Valley AIDS Council Cohort, SBP, Systolic blood pressure; DBP, Diastolic blood pressure; Abs, Absolute values*.

### Association Between ART Regimens and T2D in VACC

ART was associated with decreased viral load and increased CD4+ T cell counts but not with metabolic dysfunction. However, individuals on different ART regimens showed differences in the association with T2D (Table 4). Notably, we found that individuals on any combination regimen with INSTI were associated with increased risk to T2D [2.08 (95%CI 1.67, 2.6; *p* < 0.001)]. Other treatment regimens like NRTI + NNRTI and any combination regimens with PIs did not show association with T2D (Table 4). When stepwise logistic regression was used, with adjustment for sex, age, BMI, virus load, and treatments to explain the presence of cardiometabolic risk factors (dichotomous variables described in the method section), T2D was associated with viral loads >1,000 [OR: 1.56 (95%CI: 1.09, 2.22), *p* = 0.014], and CD4 lower than 500 [OR: 1.36 (95%CI: 0.96, 1.93), *p* = 0.088]. Any treatment with INSTI-containing combination was associated with LDL-C [OR 0.62 (95%CI: 0.48, 0.80), *p* < 0.001], systolic blood pressure [OR 0.71 (95% CI: 0.54, 0.95), *p* = 0.021] and, triglyceride concentration > 150 mg/dL [OR: 0.85 (95%CI 0.67, 1.07)].

**Table 4:** Treatments used and association with T2D in the cross-sectional analysis of VACC^¶^.

**Treatment**	**T2D**	**No T2D**	**OR (95%CI)**	***p*-value**
Any INSTI/other	510/190 (56)	408/317 (44)	2.08 (1.67, 2.05)	<0.001
Any PI/other	36/664 (51)	34/691 (49)	1.1 (0.69, 1.76)	0.692
NRTI+NNRTI/other	45/655 (46)	52/673 (54)	0.89 (0.59, 1.34)	0.577

¶ *VACC, Valley AIDS Council Cohort; INSTI, Integrase Strand Transfer Inhibitor; PI, Protease Inhibitors; NRTI, Nucleoside/Nucleotide Reverse Transcriptase Inhibitors; NNRTI, Non-Nucleoside Reverse Transcriptase Inhibitors*.

### Comparative Analysis of Metabolic Traits in the VACC and Other MA Populations

We have limited our comparisons between cohorts to the currently available data from the VACC. Both HHANES and SAMAFS have a higher proportion of female participants when compared to VACC. The mean (SD) differences between the VACC, HHANES and SAMAFS individuals in the traits shown below are: BMI, 29.4 (6), 30.9 (6), and 31.1 (7) (*p* < 0.001); triglycerides, 176 (121), 137 (109), and 151 (140) mg/dL (*p* < 0.001); and HDL-C, 44 (13), 53 (15), 48 (14) mg/dL (*p* < 0.001), respectively. This *post hoc* analysis was done with Tukey-Kramer for pairwise comparisons, all adjusted for sex, age, and T2D. Thus, the VACC individuals had lower BMI but significantly higher triglycerides and lower HDL-C. Descriptive statistics organized by sex from SAMAFS and HHANES individuals are shown in Supplementary Tables 1, 2. Both sexes showed Cohen-*d* >0.3 for diastolic blood pressure and HDL-C, and about 0.2 for BMI and triglycerides, with men showing a lower effect than females. In SAMAFS, T2D showed effects greater than Cohen-*d* 0.5 for age, systolic blood pressure, and triglycerides; Cohen-d 0.37 for BMI and 0.24 for HDL-C. Tables 5, 6 show the descriptive statistics for these traits in both SAMAFS and HHANES individuals stratified by their T2D status.

**Table 5:** General description and stratification by T2D status of selected variables from the SAMAFS^¶^.

**Variable**	**All Individuals**		**No T2D**		**T2D**		***p*-val**
	***N***	**Mean ± SD**	***N***	**Mean ± SD**	***N***	**Mean ± SD**	
Age (years)	2,498	47.4 (17.2)	1,826	43.2 (16.3)	672	58.8 (14.3)	<0.001
BMI	2,475	31.1 (7.4)	1,814	30.4 (7.2)	661	32.9 (7.5)	<0.001
SBP (mmHg)	2,260	126.9 (19.3)	1,609	123.2 (17.1)	651	135.9 (21.5)	<0.001
DBP (mmHg)	2,260	71.6 (10.5)	1,609	71.5 (10.2)	651	71.9 (11.1)	0.398
Trigly (mg/dL)	2,433	150.9 (141.1)	1,779	134.6 (119.0)	654	195.2 (181.5)	<0.001
HDL-C (mg/dL)	2,432	47.8 (13.7)	1,779	48.8 (13.6)	653	45.3 (13.7)	<0.001

¶ *SBP, Systolic blood pressure; DBP, Diastolic blood pressure; Trigly, triglycerides. SAMAFS, San Antonio Mexican American Family Studies; SBP, Systolic blood pressure; DBP, Diastolic blood pressure; Trigly, triglycerides*.

**Table 6:** General description and stratification by T2D status of selected variables from the HHANES^¶^.

**Variable**	**All Individuals**		**No T2D**		**T2D**		***p*-value**
	***N***	**Mean ± SD**	***N***	**Mean ± SD**	***N***	**Mean ± SD**	
Age (years)	1,157	48.9 (16.7)	895	45.6 (16.4)	262	60.3 (11.8)	<0.001
BMI	1,127	30.5 (6.2)	880	29.9 (6)	247	32.4 (6.2)	<0.001
SBP (mmHg)	1,071	125 (19.5)	833	122.9 (18.7)	238	132 (20.5)	<0.001
DBP (mmHg)	1,071	71.7 (12.4)	833	72.2 (12.1)	238	70 (13.4)	0.015
Trigly (mg/dL)	1,142	169.3 (153)	895	161.1 (149)	247	199.3 (163.5)	<0.001
HDL-C (mg/dL)	1,140	49.9 (13)	893	50.6 (13.2)	247	47.1 (11.9)	<0.001

¶ *SBP, Systolic blood pressure; DBP, Diastolic blood pressure; Trigly, triglycerides. HHANES, Mexican American data from the National Health and Nutrition Examination Survey; SBP, Systolic blood pressure; DBP, Diastolic blood pressure; Trigly, triglycerides*.

**Figure 3:**
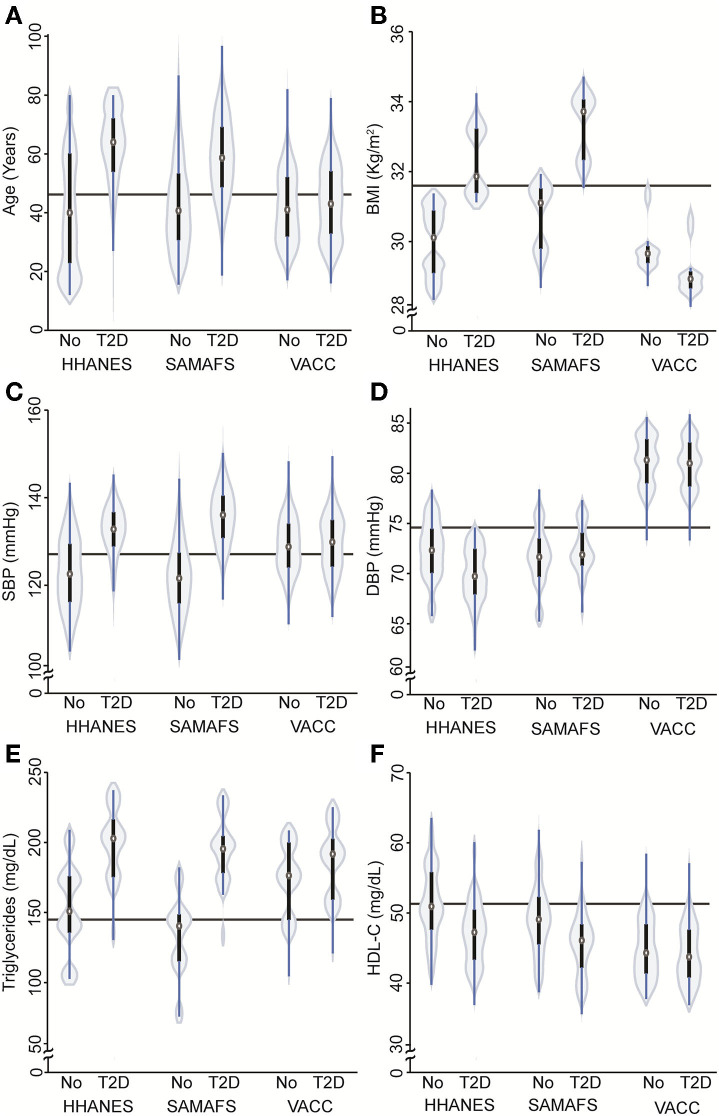
Violin plots depicting the relationship between T2D status and various demographic and cardiometabolic traits in the VACC and HIV-1 negative HHANES and SAMAFS individuals. **(A)** Violin plot showing relationship between T2D status and age in the three cohorts. Participants with T2D were older than non T2D [*b* = 13.1 (se 1.3), *p* < 0.001], but the VACC individuals showed interaction with T2D [*b* = −9.5 (se 1.9), *p* < 0.001]. **(B)** Violin plots showing relationship between age-, sex-, and cohort-adjusted BMI and T2D status in three different cohorts. Higher BMI was associated with T2D status [*b* = 2.8 (se 0.45) *p* < 0.001] in HHANES and SAMAFS, but interaction between VACC and T2D shows lower BMI when compared with other cohorts [*b* = −3.5 (se 0.55) *p* < 0.001]. **(C,D)** Violin plots showing relationship between SBP and DBP adjusted by age, sex, cohort, BMI and T2D status. For VACC, there was an interaction seen for DBP [*b* = 3.8 (se 1.0) *p* < 0.001], but not for SBP. **(E,F)** Violin plots showing relationship between triglycerides and HDL-C adjusted by age, sex, cohort, BMI and T2D status. T2D was associated with higher triglycerides concentrations [*b* = 28.2 (se 12.3), *p* = 0.02] and lower HDL-C [*b* = −3.5 (se 0.9) *p* < 0.001]. The HDL-C showed interaction for VACC [*b* = 2.2 (se 1.1) *p* = 0.042]. HHANES: The Hispanic component of NHANES 2018 cohort; SAMAFS, San Antonio Mexican American Family Studies; VACC, Valley AIDS Council Cohort. The continuous horizontal line represents the mean value without adjustment.

**Figure 4:**
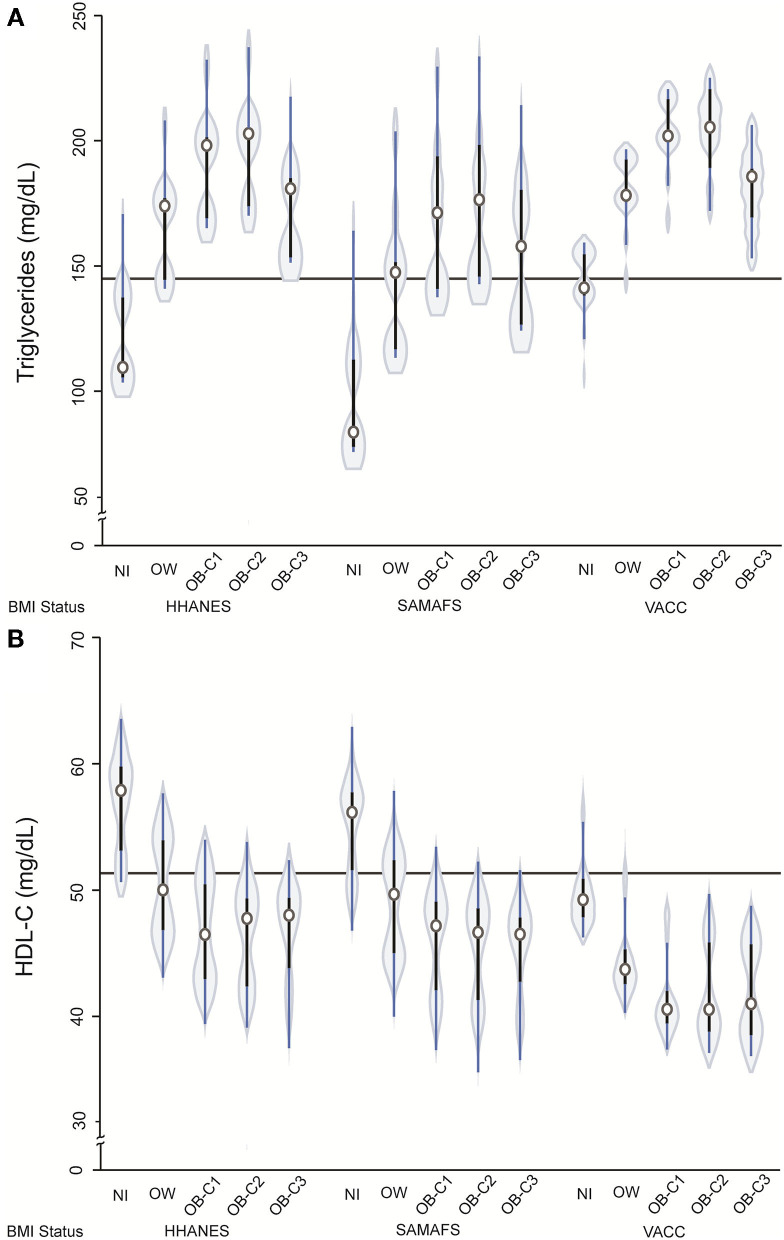
Violin plots showing relationship between triglycerides **(A)** and HDL-C **(B)** with BMI categories after adjusting for age, sex, cohort, and T2D status. The presence of diabetes was associated with higher triglyceride concentration [*b* = 28.2 (se 12.3), *p* = 0.02] and lower HDL-C [*b* = −3.5 (se 0.9) *p* < 0.001]. The HDL-C showed interaction for VACC [*b* = 2.2 (se 1.1) *p* = 0.042]. HHANES: The Hispanic component of NHANES 2018 cohort; SAMAFS, San Antonio Mexican American Family Studies; VACC, Valley AIDS Council Cohort. NW, normal weight; OW, overweight; OB-C1, obesity-category 1; OB-C2, obesity-category 2; and OB-C3, obesity category 3. The continuous horizontal line represents the mean value without adjustment.

The prevalence of T2D in VACC was 51% (i.e., 917/1,807) compared to 27% (i.e., 672/2,498) in SAMAFS and 23% (i.e., 262/1,157) in HHANES (*p* < 0.001). The males in the VACC had a greater risk of developing T2D, which is similar to that seen in the SAMAFS. However, the PWH with T2D in the VACC were younger (4.7 years or 0.3 standard deviations) and had lower BMI (BMI 2.43 units less or 3.7 standard deviations) when compared to SAMAFS. When comparing the T2D groups and adjusting for sex and pedigree structure, individuals in the VACC were younger and had lower BMI, *p*-value <0.0001 (Table 7 and Figure 3) but showed the highest DBP levels and lowest HDL-C concentrations (Figure 3). The relationship between the triglycerides and BMI showed the classic inverted U-shape relationship in all three cohorts. However, the HDL-C levels decreased by the BMI strata and were found to be lowest for the VACC individuals (Table 7 and Figure 4).

**Table 7:** General linear models for age, BMI, blood pressure, triglycerides and HDL-C.

**Variables**	**Age**	**BMI**	**SBP (mmHg)**	**DBP (mmHg)**	**Triglycerides (mg/dL)**	**HDL-C (mg/dL)**
SAMAFS^¶^	−2.61 (0.67)^**^	0.31 (0.26)^*^	0.67 (0.67)	−0.52 (0.47)	−24.38 (5.59)^**^	−2.05 (0.49)^**^
HIV	−2.75 (0.72)^**^	0.50 (0.31)	6.27 (0.71)^**^	7.63 (0.49)^**^	1.1 (6.87)	−3.11 (0.62)^**^
T2D	14.7 (0.92)^**^	2.79 (0.45)^**^	1185 (1.43)	−3.87 (0.95)^**^	28.48 (12.19)^*^	−3.47 (0.84)^**^
SAMAFS ^*^T2D	0.95 (1.13)	0.06 (0.55)	4.208(1.66)^*^	3.10 (1.0)^**^	22.57 (14.0)	−0.25 (1.01)
VACC^*^T2D	−13.12 (1.12)^**^	−3.51 (0.55)^**^	−0.95 (1.57)	3.75 (1.0)^**^	−12.71 (14.21)	2.24 (1.08)^*^
Male	–	−1.77 (0.20)^**^	4.31 (0.49)^**^	4.19 (0.31)^**^	32.73 (4.72)^**^	−6.98 (0.37)^**^
Age	–	−0.02 (0.03)^*^	0.47 (0.02)^**^	0.04 (0.01)^**^	0.11 (0.11)	0.10 (0.01)^**^
OW	–	–	3.29 (0.64)^**^	3.26 (0.40)^**^	39.3 (5.29)^**^	−5.45 (0.57)^**^
OB-Cl	–	–	4.95 (0.68)^**^	5.31 (0.43)^**^	58.27(6.25)^**^	−8.61 (0.58)^**^
OB-C2	–	–	6.39 (0.82)^**^	5.51 (0.63)^**^	66.05 (8.16)^**^	−9.22 (0.68)^**^
OB-C3	–	–	9.69 (0.95)^**^	6.50 (0.63)^**^	47.07 (6.7)^**^	−9.41 (0.74)^**^
Const	46.27 (0.584)^**^	31.6 (0.391)^**^	95.47 (0.95)^**^	64.99 (0.71)^**^	101.30 (5.91)^**^	55.34 (0.78)^**^

¶ *SAMAFS, San Antonio Mexican American Family Studies; VACC, Valley AIDS Council Cohort; OW, Overweight; OB- Cx: Obesity categories 1–3. Data are shown as beta coefficients (se)*.

* *p < 0.05*,

** *p < 0.01*.

## Discussion

Our results suggest that the PWH in the RGV developed T2D at a younger age when compared to HIV-negative individuals in the SAMAFS and HHANES. There were striking differences between the cohorts: the PWH in VACC were younger and had lower BMI but had a higher prevalence of metabolic problems when compared to individuals in HHANES and SAMAFS. Furthermore, the increased T2D risk in PWH was independent of BMI. Patients with better virological control, irrespective of the treatment regimen, had a more favorable metabolic profile.

Previous studies have shown that insulin resistance and T2D prevalence are increased in individuals who have HIV-1 infection (3). However, there are only limited studies that have examined the prevalence of HIV and T2D in the RGV region. A recent analysis by De La Garza et al. showed that the average HIV incidence rates increased by 115% per 100,000 within the RGV from 2007 to 2015, which is higher than other Texas counties with large populations (26). RGV region also has a high prevalence of adult T2D and has been estimated to be as high as 30.7% compared to a nationwide prevalence of 12.3% (8, 9). Remarkably, Restrepo et al. found that the prevalence of T2D in individuals affected with tuberculosis in the Cameron and Hidalgo counties to be 39% (27). The presence of preexisting metabolic diseases and the *de novo* metabolic complications due to HIV-1 infection and ART could contribute to unusually high T2D prevalence seen in the VACC.

While recently introduced ART regimens have greatly improved metabolic outcomes, there have been reports of persistent metabolic dysfunction in PWH (28). For example, a longitudinal study conducted by Gomes et al. suggests that ART may increase T2D risk in Hispanics (25). They examined the development of new-onset impaired fasting glucose (IFG) following ART initiation in PWH in a Dominican HIV Hispanic cohort who were initiating ART (*N* = 153; ≤ 180 days prior to starting ART). At baseline, they found that 6% had T2D and 16% had IFG. Strikingly, in this cohort, 46 developed IFG after 329/1,000 person-years follow up. Most of the individuals developed metabolic complications within 12 months following the ART start, although only 13% were on PIs. Recent data also suggests that ART regimens that are initiated with INSTIs or PIs may confer a greater T2D risk, and this may be mediated through weight gain (29). They also reported that this effect is more pronounced in raltegravir-based initiators when compared to those based on NNRTIs. Our findings in VACC suggest that any regimen containing INSTI was associated with an increased prevalence of T2D. However, in the absence of longitudinal data, we cannot attribute this increased prevalence directly to a specific regimen. Recent studies have shown that INSTI-based regimens can increase T2D risk through multiple mechanisms, including an increased deposition of central adipose tissue and adverse effects on adipose tissue that may result in insulin resistance (30, 31). While some PI-based regimens have been associated with deleterious effects on insulin secretion (32), the impact of the integrase inhibitors on insulin secretion is not well-understood. It has been speculated that INSTIs may disrupt insulin secretion by interacting with magnesium ions (33) that are critical for electrical activity and insulin secretion in pancreatic beta-cells (34). Thus, it is imperative to conduct well-controlled longitudinal studies to understand the extent of metabolic dysfunction associated with various treatment regimens in the VACC to implement individualized preventative measures to reduce future cardiometabolic risk.

Obesity is thought to be associated with a chronic inflammatory state in the adipose tissue which is mediated through a number of inflammatory signals/cells [e.g., M1 macrophages, inflammatory cytokines (TNF-alpha and IL-6), and activated CD4+ and CD8+ T cells] (35), HIV/ART further exacerbate this condition through further modulation of inflammatory cytokines and mitochondrial dysfunction (36). Prior to the introduction of the newer ART regimens, lipodystrophy was a common feature in HIV-infected persons (37, 38). In contrast, recent studies have shown the increasing incidence of obesity and overweight in this group, which can be as high as 70% (39–43). In contrast to these findings, VACC individuals had a lower mean BMI when compared to individuals enrolled in SAMAFS, HHANES and RGV-based cohorts or their subsets (9, 44). However, in contrast to some RGV-based HIV-negative cohorts (9), we did not find a difference in the obesity prevalence in the diabetic and non-diabetic individuals in the VACC. Hernandez-Romieu et al. also found similar trends in the NHANES individuals. Using 2009–2010 data, they found that diabetes prevalence is much higher in HIV-infected adults when compared to the general U.S. adult population in the absence of obesity (45). Also, they also found that HIV-infected individuals had a higher prevalence of diabetes at younger ages. This higher T2D prevalence in PWH may be related to a chronic inflammatory state induced by HIV-1 infection and ART-induced insulin resistance.

Recent studies have suggested that the major classes of ART have differential effects on weight gain (31, 46–50) and abnormalities in fat and lipid storage (51). For example, Menard et al., reported that there was a mean weight gain of ~3 kg in a cohort (*N* = 462) receiving an integrase inhibitor-based ART regimen (dolutegravir) after a follow-up of an average of 276 days (46). Using a large study population (ACTG A5257 clinical trial; *N* = 1,809) in which the treatment naïve HIV were randomized into three different treatments: raltegravir (integrase inhibitors) or atazanavir/ritinovir (PIs) or darunavir/ritonavir (PIs), all in combination with tenofovir disoproxil fumerate/emtricitabine, Bhagwat et al., reported that blacks on integrase inhibitors had larger increases in waist circumference when compared to non-blacks (31). In the same study, they found that women had a greater increase in waist circumference when compared to men. In the VACC, we could not find any relationship between obesity prevalence and the broad ART regimens, and future longitudinal studies are needed to understand this phenomenon. Remarkably, we found that increasing BMI had a positive association with absolute CD4+ cell counts. This finding is in agreement with a previous study that also showed a positive relationship between higher BMI and CD4+ cell recovery in HIV-1 infected people on ART (52). Notably, such a positive relationship between higher BMI levels and CD4+ counts is also described in the absence of HIV-1 infection (53). The relevance of such an increase in CD4+ counts with increasing BMI in PWH needs additional studies.

Our study has some important limitations that are commonly encountered when extracting data from clinical records, including missing data, unavailability of records that are relevant for metabolic diseases such as waist circumference, physical activity, dietary habits, acculturation factors, co-infection with HCV, mode of disease transmission, prediabetes, among others. An important limitation is that we have relied on BMI as a major determinant to assess metabolic health in the VACC individuals in absence of other more appropriate obesity measures. While body composition measures such as waist circumference and fat mass would be more suitable to assess metabolic dysfunction in non-obese PWH due to HIV and ART-induced fat alterations, BMI categories have been successfully used to evaluate metabolic health in absence of such measures. For example, Lake et al. showed that in the Multicenter AIDS Cohort Study (MACS) normal weight and overweight HIV-positive men are much less likely to be metabolically healthy when compared to non-obese HIV-negative men (54). Given that the recent reports suggest that integrase inhibitor-based regimens could result in increased accumulation of both subcutaneous and visceral adipose tissue (55), we plan to conduct additional assessments for body composition in the VACC participants for future studies. Another limitation of our study is that the data were available for only 20 months due to a recent switch from a different electronic medical records database. Future studies will incorporate these additional factors and longitudinal designs to gain further insights into T2D pathogenesis in the VACC. Nevertheless, our analyses recapitulated several key aspects of HIV disease pathogenesis, including an inverse relationship between viral load and CD4+ cell count and CD4/CD8 ratio, thus suggesting our cross-sectional data are highly informative.

In summary, the prevalence of T2D in the PWH in the Rio Grande Valley is higher than the national and South Texas Hispanic populations. Remarkably, this increased prevalence is not associated with a higher BMI, suggesting that factors independent of obesity may be in part responsible for disease pathogenesis. Despite their lower BMI, these individuals showed increased dyslipidemia and hypertension, reflecting poor metabolic health. Further cross-sectional and longitudinal studies are necessary to develop personalized interventions to improve metabolic health and reduce future cardiovascular-related morbidity and mortality.

## Data Availability Statement

Publicly available datasets were analyzed in this study. This data can be found here: https://wwwn.cdc.gov/nchs/nhanes/continuousnhanes/default.aspx?BeginYear=2017 and dbGaP Study Accession: phs000847.v1.p1.

## Ethics Statement

The studies involving human participants were reviewed and approved by Institutional Review Board, University of Texas Rio Grande Valley. Written informed consent for participation was not required for this study in accordance with the national legislation and the institutional requirements.

## Author Contributions

JL-A, DAM, RD, SM, and RDM contributed to the study conception and study design. DAM and RDM were responsible for the clinical care of the PWH and supervised the collection of clinical data from VACC. SAMAFS data was collected under the supervision of DL, RD, JEC, and JB. JL-A performed the statistical analysis with contributions from AD-B, SM, and RD. AD-B, LM, RA, and CPJ contributed to the data analysis and interpretation of the data. JL-A wrote the initial draft of the manuscript, which was critically revised by DAM, RD, SM, and RDM. All authors contributed to the article and approved the submitted version.

## Conflict of Interest

The authors declare that the research was conducted in the absence of any commercial or financial relationships that could be construed as a potential conflict of interest.

## Supplementary Material

Supplementary Figure 1 Histograms showing the distribution of raw variables **(A)** and after transformation **(B)**. The frequency of CD4 absolute counts is in the upper panel. The lower panel shows the frequency of the squared root variable. The transformation resulted in an approximately normal distribution.

Supplementary Table 1 General description and stratification by sex of selected variables from the SAMAFS^¶^.

Supplementary Table 2 General description and contrast by sex of selected variables from HHANES^¶^ 2018 data.
