# Supplementary figures and images for: Association of HIV-1 Infection and Antiretroviral Therapy With Type 2 Diabetes in the Hispanic Population of the Rio Grande Valley, Texas, USA
Source: Front Med (Lausanne). 2021 Jul 5;8:676979. doi: 10.3389/fmed.2021.676979 (PMC8287129; doi:10.3389/fmed.2021.676979)

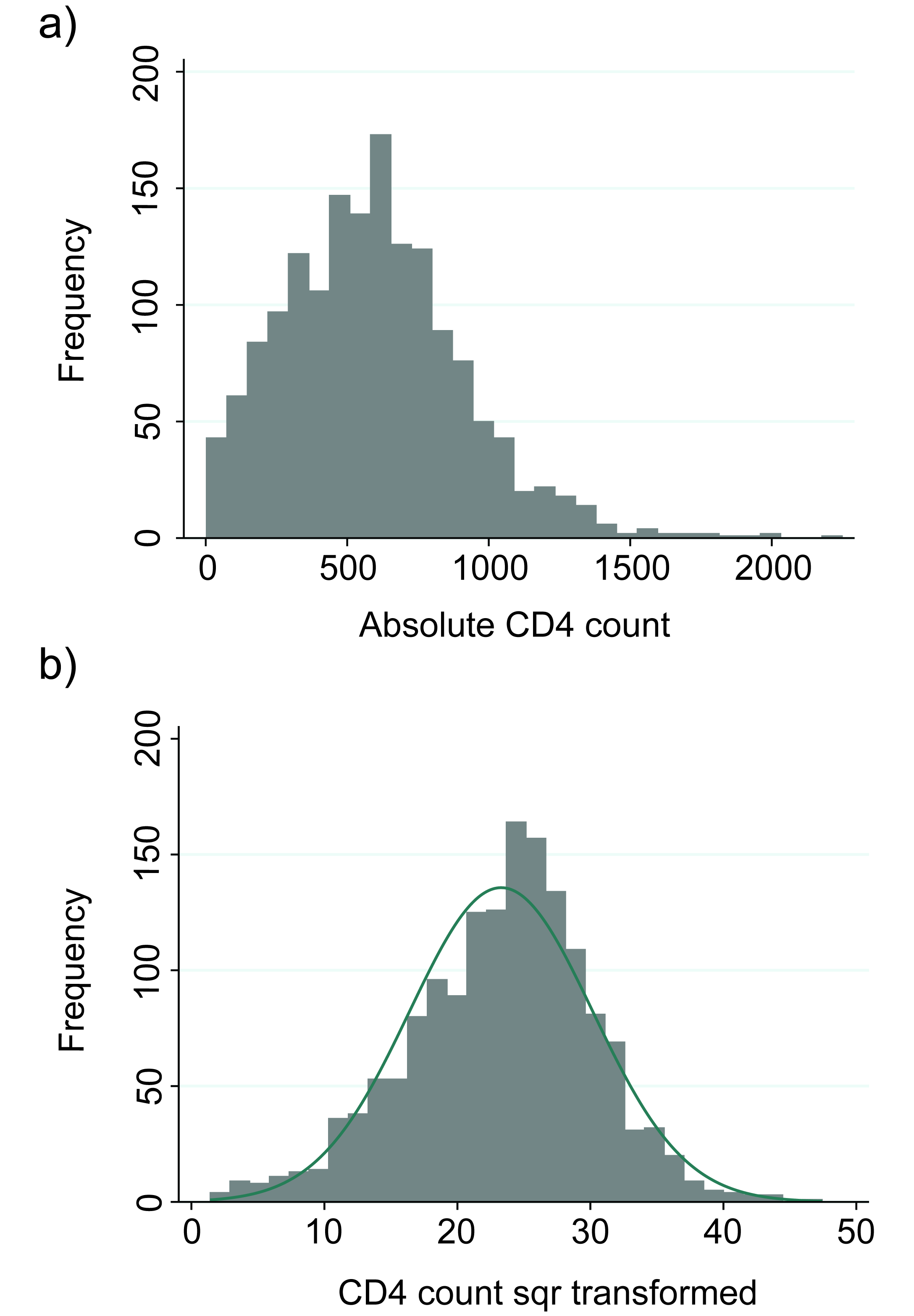

Supplement: Supplementary Figure 1 — Histograms showing the distribution of raw variables (A) and after transformation (B). The frequency of CD4 absolute counts is in the upper panel. The lower panel shows the frequency of the squared root variable. The transformation resulted in an approximately normal distribution. [file Image_1.TIF]
