# Supplementary material for: Association of HIV-1 Infection and Antiretroviral Therapy With Type 2 Diabetes in the Hispanic Population of the Rio Grande Valley, Texas, USA
Source: Front Med (Lausanne). 2021 Jul 5;8:676979. doi: 10.3389/fmed.2021.676979 (PMC8287129; doi:10.3389/fmed.2021.676979)
Supplement: Supplementary Table 1 — General description and stratification by sex of selected variables from the SAMAFS¶. [file Table_1.docx]

**Supplementary Table 1.** General description and stratification by sex of selected variables from the SAMAFS^¶^.

| **Variable** | **All Individuals** | | **Females** | | **Males** | | **p-val** |
| --- | --- | --- | --- | --- | --- | --- | --- |
|  | **N** | **Mean±SD** | **N** | **Mean±SD** | **N** | **Mean±SD** |  |
| Age (years) | 2498 | 47.4 (17.2) | 1475 | 48.0 (17.0) | 1023 | 46.5 (17.5) | 0.029 |
| BMI | 2475 | 31.1 (7.4) | 1463 | 31.6 (7.9) | 1012 | 30.0 (6.4) | <0.001 |
| SBP (mmHg) | 2260 | 126.9 (19.3) | 1335 | 126.0 (21.0) | 925 | 128.1 (16.6) | 0.01 |
| DBP (mmHg) | 2260 | 71.6 (10.5) | 1335 | 70.1 (10.0) | 925 | 74.9 (10.7) | <0.001 |
| Trigly (mg/dL) | 2433 | 150.9 (140.1) | 1437 | 138.4 (95.3) | 996 | 138.4 (95.3) | <0.001 |
| HDL-C (mg/dL) | 2432 | 47.8 (13.7) | 1439 | 50.2 (13.4) | 993 | 44.4 (13.4) | <0.001 |

^¶^SAMAFS, San Antonio Mexican American Family Studies; SBP, Systolic blood pressure; DBP, Diastolic blood pressure; Trigly, triglycerides.
