# Supplementary material for: Association of HIV-1 Infection and Antiretroviral Therapy With Type 2 Diabetes in the Hispanic Population of the Rio Grande Valley, Texas, USA
Source: Front Med (Lausanne). 2021 Jul 5;8:676979. doi: 10.3389/fmed.2021.676979 (PMC8287129; doi:10.3389/fmed.2021.676979)
Supplement: Supplementary Table 2 — General description and contrast by sex of selected variables from HHANES¶ 2018 data. [file Table_2.docx]

**Supplementary Table 2.** General description and contrast by sex of selected variables from HHANES^¶^ 2018 data.

| **Variable** | **All Individuals** | | **Female** | | **Male** | | **p-value** |
| --- | --- | --- | --- | --- | --- | --- | --- |
|  | **N** | **Mean±SD** | **N** | **Mean±SD** | **N** | **Mean±SD** |  |
| Age (years) | 1252 | 48.6 (16.8) | 664 | 48.8 (16.5) | 588 | 48.3 (17.1) | 0.631 |
| BMI | 1168 | 30.4 (6.2) | 619 | 30.7 (6.8) | 549 | 30.1 (5.4) | 0.141 |
| SBP (mmHg) | 1108 | 124.9 (19.6) | 592 | 123.5 (21.2) | 516 | 126.6 (17.3) | 0.01 |
| DBP (mmHg) | 1108 | 71.8 (12.4) | 592 | 69.8 (12.6) | 516 | 74.1 (11.8) | <0.001 |
| Trigly (mg/dL) | 1142 | 169.3 (153) | 610 | 146.4 (89.7) | 532 | 195.6 (199.4) | <0.001 |
| HDL-C (mg/dL) | 1148 | 49.9 (13.1) | 614 | 53.9 (13.1) | 534 | 45.2 (11.4) | <0.001 |

^¶^HHANES, Mexican American data from the National Health and Nutrition Examination Survey; SBP, Systolic blood pressure; DBP, Diastolic blood pressure; Trigly, triglycerides.
